# Acute Promyelocytic Leukemia in a Woman with Thalassemia Intermedia: Case Report and Review of Literature on Hematological Malignancies in β-Thalassemia Patients

**DOI:** 10.3390/hematolrep14040045

**Published:** 2022-10-21

**Authors:** Claudio Pellegrino, Giulia Dragonetti, Patrizia Chiusolo, Monica Rossi, Nicoletta Orlando, Luciana Teofili

**Affiliations:** 1 Dipartimento di Diagnostica per Immagini, Radioterapia Oncologica ed Ematologia, Fondazione Policlinico Universitario “A. Gemelli” IRCCS, I-00168 Roma, Italy; 2 Sezione di Ematologia, Dipartimento di Scienze Radiologiche ed Ematologiche, Università Cattolica del Sacro Cuore, I-00168 Rome, Italy

**Keywords:** acute promyelocytic leukemia, β-thalassemia, hematological malignancies, retinoic acid, ATRA differentiation syndrome

## Abstract

Patients affected by transfusion-dependent β-thalassemia are prone to developing several clinical complications, mostly related to the iron overload. We report the case of a patient affected by transfusion-dependent β-thalassemia (TDT) developing acute promyelocytic leukemia (APL). In our case, the therapeutic management was significantly complicated not only by myocardial dysfunction, but also by the occurrence of the differentiation syndrome following all-trans retinoic acid (ATRA) administration. We carried out a careful revision of the current literature on the occurrence of hematological malignancies in β-thalassemia patients to investigate the major complications so far described. APL occurrence in β-thalassemia patients has been very rarely reported, and our experience suggests that TDT patients suffering pre-existing comorbidities may develop a potentially fatal complication during ATRA therapy.

## 1. Introduction

β-thalassemia syndromes are a group of hereditary disorders characterized by a genetic deficiency in the synthesis of β-globin chains, resulting in accumulation and deposition of unpaired α-globin chains in red blood cells (RBCs). This causes hemolysis and ineffective hematopoiesis leading to chronic anemia. Currently, the availability of proper care and appropriate treatment strategies, including regular transfusions, allogeneic stem cell transplantation, inducers of fetal hemoglobin synthesis, and iron chelation therapy, have resulted in increased life expectancy and quality of life in patients suffering from β-thalassemia major. The prolonged life span increases the possibility that other diseases such as malignancies may occur, possibly due to the iron-overload-induced oxidative stress in bone marrow microenvironment, and the increased hematopoietic drive [1]. In this study we report the case of a patient affected by transfusion-dependent β-thalassemia developing acute promyelocytic leukemia (APL).

In our case, complications related to the β-thalassemia significantly hampered the management of the APL treatment. Therefore, we carefully reviewed current literature to investigate whether other authors had previously reported APL occurrence in this setting. Moreover, we explored whether the underlying β-thalassemia affected the therapy management of hematological malignancies, finally worsening the prognosis of these patients.

## 2. Case Report

A 69-year-old woman was tracked as an outpatient for a β-thalassemia intermedia, due to the HBB gene heterozygous mutation c.92 + 1G > A (rs33971440, VAF 48.73) and a duplication triple α anti 3.7 (ααα^anti3.7^). At her first visit to our hospital, she was aged 49 years and had been receiving occasional transfusions, mainly in association with concomitant infectious episodes. She was positive for both HBV and HCV hepatitis viruses. The patient was gradually shifted to a regular transfusion regimen for progression of splenomegaly, worsening anemia, and appearance of formations compatible with extra-medullary hematopoiesis (EMH) in the para-vertebral region (38 × 17 mm). She was given iron chelation therapy, first with deferoxamine and then with deferasirox. Since MRI for iron overload estimation was not available at our hospital, the patient had no quantitative assessment, and iron chelation therapy was administered according to ferritin levels.

The transfusion management was complicated by the development of anti-E allo-immunization and cold agglutinins. The patient was treated for HBV infection with Entecavir and for HCV infection with direct-acting antivirals: at last hepatology visit, an HBV-related hepatic cirrhosis was documented (liver stiffness 9.7 kPa, Metavir F3) with portal hypertension (mild thrombocytopenia without ascites or history of upper gastrointestinal bleeding). She was also suffering from asthma-chronic pulmonary obstructive disease (CPOD) overlap syndrome and bronchiectasis. Moreover, she had an episode of non-valvular atrial fibrillation in 2018, and was successfully subjected to cardioversion with amiodarone. Other significant comorbidities included osteoporosis with a history of bilateral wrist fractures, recurrent superficial thrombophlebitis, and anxious-depressive syndrome.

**Figure 1:**
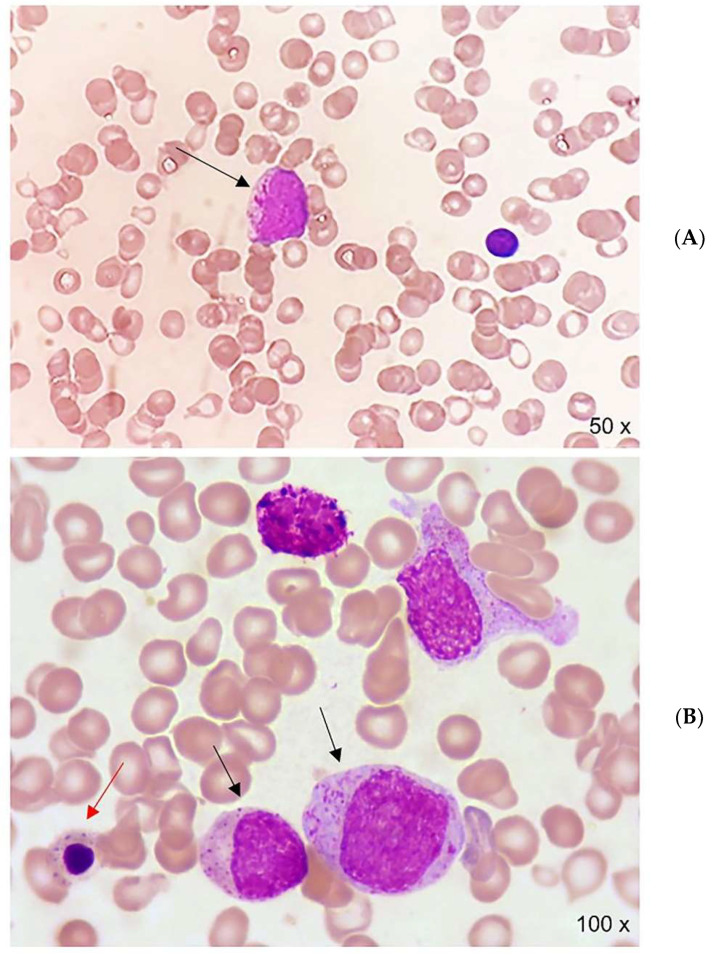
(**A**) ***Upper picture*:** peripheral blood smear, showing anisopoikilocytosis, microcytic hypochromic red cells, and numerous target cells. In the center, a leukemic promyelocyte (black arrow). (**B**) ***Lower picture*:** bone marrow blood smear, showing promyelocytes (black arrows) and marked dyserythropoiesis (red arrow).

In May 2021, the patient underwent total knee arthroplasty surgery and was subsequently admitted to a rehabilitation ward. During the post-operative course, the blood cell count (CBC) showed a progressive decrease in platelet and neutrophil count. The CBC at nadir documented WBC 3.4 × 10^9, platelets 24 × 10^9/L, with the smear examination showing 34% of lymphocytes, 6% of monocytes, 26% of neutrophils, 5% of granulocyte precursors, and 27% blast cells. A marked dysplasia of the granulocytic series was also documented. Blast morphology was reminiscent of abnormal promyelocytes [2] (Figure 1A). Physical examination was unremarkable except for the presence of palpable hepato-splenomegaly. Bone marrow blood smear showed a marked (56%) infiltration of myeloid peroxidase positive blast and abnormal promyelocytes (Figure 1B). Flow cytometry reported a blastic myeloid population CD117 + CD34 +/− CD13 + CD33 ++ HLA–DR− accounting for 71% of the total bone marrow cellularity. Molecular analysis with nested RT–PCR revealed a bcr3 isoform of the promyelocytic leukemia/retinoic acid receptor alpha (PML–RARA) transcript and a FLT3 ITD.

Based on the aforementioned exams, diagnosis of intermediate risk acute promyelocytic leukemia was made [3] and the patient started an induction therapy with all-trans retinoic acid (ATRA) 60 mg/die and idarubicin 7.45 mg (5 mg/m^2^) for four consecutive days, in association with methylprednisolone 20 mg/die for the prevention of the differentiation syndrome [4].

**Figure 2:**
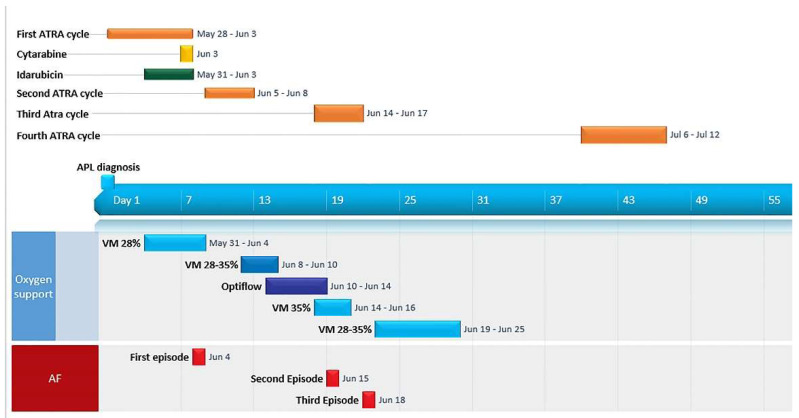
Gantt graph displaying patient’s clinical course during the treatment of acute promyelocytic leukemia (Office Timeline.Version 6.00.06, 2021 Office Timeline Inc., Bellevue WA 98004, USA). **AF**: atrial fibrillation; **ATRA**: all trans-retinoid acid; **VM**: Ventimask.

**Figure 3:**
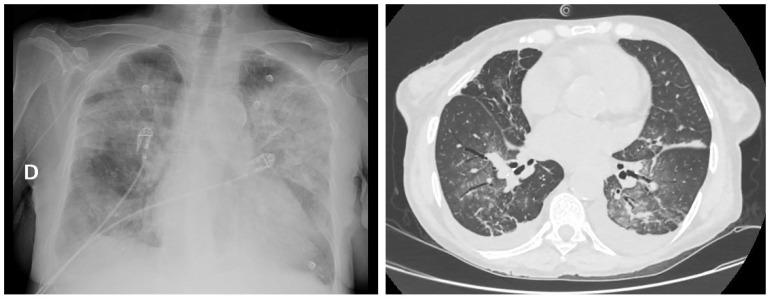
Chest X-ray and TC scan documenting bilateral pulmonary infiltrates and pleural effusion during ATRA differentiation syndrome (Carestream Vue Motion Version 12.1.5.9529 © Carestream Health, Inc., Rochester, NY, USA, 2021). D: RIGHT side.

The patient’s clinical course is summarized in Figure 2. Starting from the fourth day of ATRA administration, we assisted a rise in WBC count, associated with the development of peripheral edema, dyspnea and oxygen desaturation. A chest X-ray revealed bilateral pulmonary infiltrates and an enlarged cardiac shadow with pleural effusion (Figure 3). On the suspicion of ATRA differentiation syndrome (DS), ATRA was temporarily discontinued, and two boli of cytarabine 500 mg were administered to rapidly lower the WBC count; moreover, methylprednisolone was switched to dexamethasone 20 mg/die, and oxygen supplementation was started. Clinical management was further complicated by the development of atrial fibrillation with rapid ventricular response, successfully cardioverted with amiodarone.

On day 9, given the improvement in clinical condition and the lowered peripheral WBC count, ATRA therapy was resumed at full dosage. During the subsequent three days, the patient’s condition worsened, with dyspnea, respiratory rate up to 30 breaths per minute, recurrent episodes of hemoptysis and peripheral blood oxygen desaturation. A bronchoalveolar lavage (BAL) was achieved, positive for Pseudomonas Aeruginosa and CMV genome (4445 copies/mL). Retinoic acid was suspended, and ventilatory support with nasal high flow therapy (Optiflow™, 60 L/min) was started, in association with diuretic (furosemide in continuous infusion) and an antimicrobial therapy (meropenem, amikacin, liposomal amphotericin B and foscarnet). The progressive improvement of pulmonary function prompted the reintroduction of ATRA on day 18 (Figure 2). Even this third attempt was unsuccessful, as during the subsequent days the patient rapidly developed signs of severe differentiation syndrome, including weight gain of up to 20 kilos, acute renal failure, and dyspnea with radiological evidence of bilateral pulmonary infiltrates, consolidation, and pleural effusion. The clinical course was further complicated by three episodes of atrial fibrillation, all successfully managed with pharmacological cardioversion. Liquid restriction, diuretic therapy, albumin replacement, ATRA discontinuation and dexamethasone produced a clear benefit. On day 40, ATRA therapy at lower doses (30 mg/day) was finally restarted. On day 47 the patient was finally dismissed.

The bone marrow examination performed on day 70 displayed a complete morphological response and the absence of the PML/RAR-α transcript was documented. Due to several comorbidities, no further chemotherapy was administered, and the patient was offered a palliative care program.

## 3. Systematic Review of Studies on β-Thalassemia and Hematological Malignancies

Hepatic cancer is the neoplasia most frequently occurring in β-thalassemia patients due to the liver iron overload. Hematological neoplasms and other solid cancers have seldom been reported and attributed to the chronic stress in the bone marrow and to iron overload [1]. We conducted a systematic review on the occurrence of hematological malignancies in β-Thalassemia, with a special focus on acute leukemia. The reporting of this systematic review was guided by the standards of the Preferred Reporting Items for Systematic Review and Meta-Analysis (PRISMA) statement, when applicable.

We performed a systematic search on the PubMed database using the following queries: “β-Thalassemia” [Mesh] AND “Neoplasms” [Mesh]. No additional search filters were applied. C.P and L.T independently controlled all references, including case reports, case series, and reviews. Discrepancies were discussed and resolved together.

Up to June 2021, **252** total references were identified. We excluded papers reporting solid neoplasm and extra-medullary hematopoietic masses mimicking solid neoplasms, papers reporting secondary neoplasms after hematopoietic stem cell transplantation, papers reporting patients with hemoglobinopathies other than β-thalassemia/HbE, communications at congresses, papers reporting hematological neoplasms in β-Thalassemia minor, duplicated studies, and papers with an abstract not in English. In the end, 25 papers were included in the review. Collected data included study type, patient characteristics, type and clinical presentation of hematological malignancy, therapy, and outcome. Results are shown in Table 1.

## 4. Discussion

The low number of cases so far described suggests that β-thalassemia does not predispose patients to hematological malignancies: the most frequent reported was NHL, strictly followed by HL, ALL, and MPN. Whether the occurrence of these hematological malignancies is the result of some kind of interaction rather than a pure coincidence remains speculative: iron, transfusion-transmitted viruses, immunosuppression secondary to blood transfusion, and hematopoietic drive can all theoretically contribute to an increase in this risk [30]. A nationwide retrospective cohort study from Taiwan showed that patients with thalassemia exhibited a 5.32-fold higher risk of developing lymphoma or leukemia than the control cohorts. In particular, transfusion-dependent thalassemia patients were 9.31-fold more likely to develop a hematologic malignancy than non–transfusion-dependent patients [31]. However, only large international multicenter studies including patients treated according to current standards and accessing novel therapies could definitively clarify this issue.

It is conceivable that especially elderly β-thalassemia patients, not treated according to current therapy standards, may develop numerous complications, including cardiopulmonary dysfunction, liver diseases, splenomegaly, endocrinopathies, and thromboses. All these complications may confound the diagnosis and hamper clinical management and treatment of the associated hematological neoplasia. The diagnosis of a hematological malignancy appears to be challenging in this peculiar population: for example, polycythemia vera tends to be masked because of the antagonizing hematological effects, one blunting the effect of the other. Moreover, the presence of some overlapping features between β-thalassemia and malignancies such as a pre-existing splenomegaly, anemia, fatigue, and infection susceptibility tends to delay diagnosis, thus worsening prognosis. Importantly, there was no significant difference in the reported toxicity profile of chemotherapy in comparison to non-thalassemic patients, except for a higher need of packed red blood cell transfusions [6].

To the best of our knowledge there is only one report in literature of a concomitant diagnosis of β-thalassemia and APL: a 32-year-old man affected by transfusion dependent HCV positive β-thalassemia major, complicated by iron overload-related cardiomyopathy, who developed a low-risk APL with t(15;17) [21]. He achieved hematological complete remission with persisting PML/RAR-α positivity after an induction course with ATRA and idarubicin: unfortunately, he died of cardiac complications. In an analogy to our case, the chemotherapy-sensitive myocardium status in thalassemia suggest caution in the use of cardiotoxyc drugs (antracycline, arsenic trioxide) in this fragile population, with a complete baseline cardiac function evaluation and a strict monitoring during therapy. Accordingly, our patient was deemed unfit to receive arsenic trioxide despite the intermediate risk of APL.

One of the most striking features of our patient was the susceptibility to developing pulmonary disease following ATRA exposure. The DS presents with acute respiratory distress, weight gain, unexplained fever, interstitial pulmonary infiltrates, pleural/pericardial effusions, hypotension, and acute renal failure. It is reported to occur during induction in 2.5% to 31% of APL patients and is associated with 15% of induction deaths [32]. Higher peripheral blood blast counts on admission as well as higher body mass index are the most consistently reported risk factors for DS development [33]. The pathogenesis of APL DS is complex and not completely elucidated: the key effects of ATRA include the release of a variety of cytokines and pro-inflammatory mediators by differentiating blast cells and the induction of a change in adhesive properties on blast cells. The combination of a systemic inflammatory state with increased vascular permeability caused by endothelial damage results in hypotension and organ hypo-perfusion, which can ultimately lead to a multi-organ failure [34].

Basically, β-thalassemia itself is associated with endothelial activation and dysfunction, coagulation abnormalities, and chronic inflammation, all leading to arterial or venous thrombosis, pulmonary hypertension, and cerebrovascular events. [35,36]. The risk increases with aging, splenectomy, and transfusion burden [35,36]. Cellular and molecular mechanisms include red blood cells’ membrane alterations, increased platelets aggregation, and elevation in extracellular vesicles [37]. It is therefore plausible that this peculiar milieu may predispose patients to DS development, especially in pulmonary circulation, particularly susceptible to the thalassemia-induced damage. In fact, β-thalassemia patients display higher levels of markers for endothelial activation such as sICAM-1, sVCAM-1, HMGB1, and P- and E-selectins, reflecting a baseline pro-adhesive phenotype of endothelial cells [38,39,40]. Likewise, treatment with ATRA promotes a striking up-regulation of adhesion molecules on APL blast cells such as CD11b, CD11c, CD15, CD65, and CD54, thus leading to lung infiltration and inflammation [41,42,43]. Finally, there is a significant elevation of chemokine levels (CXCL1, CXCL9, CXCL10, and CXCL12) in TD β-thalassemia patients [44], while ATRA differentiation therapy can itself induce chemokine production in the lungs and chemokine receptors expression in APL cells, triggering migration of leukemic cells [45].

It should be also considered that ATRA-driven granulocytic differentiation involves the reprogramming of over 300 molecular pathways through the modulation of several transcriptional factors [46]. Among them, PU.1 is crucial for normal hematopoiesis and terminal differentiation of granulocytes, and has a well-established role in leukemia suppression [47]. PU.1 expression is suppressed in APL and restored by ATRA. Of note, dysfunctional features of granulocytes in β-thalassemia patients have been ascribed to a reduced expression of PU.1, as a result of iron overload, leading to reduced oxidative burst activity, and elevated levels of membrane lipid peroxidation [48,49]. Indeed, we could also speculate that ATRA action may be altered in β-thalassemia patients developing APL, given the pre-existing impaired myeloid terminal differentiation due to PU.1 deficiency.

## 5. Conclusions

The occurrence of hematological neoplasms in β-thalassemia patients has been linked to the chronic stress in the bone marrow, iron overload and transfusion transmitted viruses. Nevertheless, an increased risk has not yet been incontrovertible demonstrated. In any case, thalassemia related complications as cardiopulmonary dysfunction, liver disease and splenomegaly tend to hamper the diagnosis and treatment of the malignancies, especially in older patients. Here we reported a case of APL in a β-thalassemia woman: considering the rarity of this concomitance, with only another case reported in literature, the most plausible conclusion is that the two events are purely coincidently. However, this ominous duo poses a particular challenge to clinicians, as these patients may be prone to develop cardiac toxicity during therapy and ATRA DS. We therefore recommend caution in the use of cardiotoxic drug (anthracycline, arsenic trioxide), with a baseline cardiac evaluation and a strict monitoring during therapy, especially in patients with a pre-existing thalassemia-related cardiomyopathy. Moreover, attention should be paid at early signs of DS, like dyspnea, fever, weight gain and appearance of radiographic opacities. Further research is needed to shed light on the complex biology of these two diseases to improve clinical management of this population.

## Figures and Tables

**Table 1:** Studies reporting the occurrence of hematological malignancies in transfusion dependent and independent β-thalassemia patients.

Malignancy	Reference	Patients (*n*)	Sex/Age	Hemoglobinopathy	Splenectomy	Clinical Presentation	Therapy/Outcome
**HL**	Balestrazzi and Butturini,1985 [5]	1	M/10	BTM	ND	Lymphocyte rich cHL (CS II2B)	6MOPP + RT DF at 3 years
	Thapa et al., 2009 [6]	1	F/7	BTM	No	Mixed cellularity cHL; stage IV	6 COPP, Complete remission
	Jabr et al., 2006 [7]	1	M/28	BTI	No	Mixed cellularity cHL; IIIa	ABVD, complete remission
	Ahad-Aziz Qureshi et al., 2019 [8]	1	M/21	BTM	Yes	Lymphocyte-rich cHL	Not reported
**NHL**	Tozzi-Cecchetti, 1979 et al. [9]	1	M/11	BTM	No	Diffuse follicular center lymphoma	1 VMP Death at 3 months
	Schilirò et al., 1979 [10]	1	M/4	BTM	No	NHL with bone marrow and CNS involvement.	1 HOP + 2 IT-MTX + prophylactic CI. Death after relapse.
	Otrock et al., 2006 [11]	1	F/25	BTM	No	DLBCL	4 CNOP + RT. Complete remission, 6 years’ follow-up
	Ricchi et al., 2004 [12]	1	F/35	BTM, HCV+	Yes	Systemic High-grade NHL with cardiac localization	3 RTX + surgery. Death for hemopericardium with cardiac tamponade
	Triantos et al., 2013 [13]	1	ND	BTM	ND	NHL	Not reported
	Chehal et al., 2002 [14]	1	F/62	BTI	No	Large B cell lymphoma with bone marrow involvement	6 CVP + 4 RTX. Complete remission
**ALL**	Tuğcu et al., 2014 [15]	1	M/12	BTI	No	B-ALL	Chemotherapy Complete remission
	Kottaridis et al., 2000 [16]	1	M/34	BTM	No	T-ALL	Chemotherapy + alloHSCT Complete remission at 1 year
	Goyal et al., 2018 [17]	1	M/22	BTM	No	ALL in the context of HIV and HTLV-I infection	Death before treatment
	Sherief et al., 2015 [18]	1	M/11	BTM, HCV+	No	B-ALL	Chemotherapy Complete remission
	Palomo-Colli et al., 2018 [19]	1	M/6	BTM	Yes	ALL-L1, B phenotype	Chemotherapy CNS radiotherapy 7y in Complete remission
**AML**	Felici et al., 1988 [20]	1	M/6	BTM	No	AML-M4	Chemotherapy Death 10d after induction
	Romani et al., 2007 [21]	1	M/32	BTM, HCV+	No	APL	ATRA+ idarubicine (induction) ATRA + idarubicine + ara-c (consolidation) Death for liver failure
**Histiocytic disorder**	De Soccio et al., 2020 [22]	1	F/4	BTM	No	Solitary xantogranuloma of the hypopharynx and cutaneous mastocytosis	Surgery. Complete remission at 11 months
**MM**	Arora & Ganapathi, 2020 [23]	1	M/46	BTI	ND	IgG lambda myeloma	Not reported
**MPN**	Voskaridou et al., 2002 [24]	1	M/32	BTI	Yes	BCR/ABL+ CML with marked thrombocytosis	Hydroxyurea + Anagrelide
	Wu et al., 2020 [25]	1	F/24	BTI	Yes	ET	Observation
**Various**	Alavi et al., 2013 [26]	2	F/16	BTM, HCV+	No	HL nodular sclerosis, IIA	6 alternating MOPP/ABVD + RT; CR at 10y
			F/13	BTI	No	CML, progressed to AML	Imatinib, CT. Death at 3 months
	Benetatos et al., 2008 [27]	2	M/37	BTI, HCV+	Yes	Marginal zone lymphoma, IIIa	Watch and wait. Stable disease at 2y
			F/52	BTI	No	Nodular lymphocytic predominant HL IIIa	ABVD complete remission
	Karimi et al., 2009 [28]	10	M/10	BTM	Yes	cHL	Chemotherapy
			M/6	BTM	No	cHL	Chemotherapy
			M/20	BTM, HCV+	No	CML	Chemotherapy
			M/16	BTM	Yes	ALL-preB	Chemotherapy
			M/6	BTI	Yes	AML-M4	Chemotherapy
			M/12	BTI	No	ALL-T	Chemotherapy
			M/23	BTM, HCV+	Yes	DLBCL	Chemotherapy
			F/28	BTM, HCV+	Yes	ALL-T	Chemotherapy
			M/25	BTM, HCV+	Yes	NHL	Chemotherapy
			F/24	BTM	Yes	cHL	Chemotherapy
	Zurlo et al., 1989 [29]	5	ND	BTM	ND	2 NHL; 2 ALL; 1 AML	Not reported

Acute lymphoblastic leukemia (**ALL**), Acute myeloid leukemia (**AML**), Acute promyelocytic leukemia (**APL**), all trans retinoid acid (**ATRA**), Beta thalassemia major (**BTM**), beta thalassemia intermedia (**BTI**), classical Hodgkin lymphoma (**cHL**), Chronic myeloid leukemia (**CML**), Diffuse large B cell lymphoma (**DLBCL**), Essential thrombocythemia (**ET**), Hodgkin lymphoma (**HL**), Non-Hodgkin lymphoma (**NHL**), Multiple myeloma (**MM**), myeloproliferative neoplasm (**MPN**).

## Data Availability

Not applicable.

## Abbreviations

Atrial fibrillation (**AF**)**,** Acute lymphoblastic leukemia (**ALL**), Acute myeloid leukemia (**AML**), Acute promyelocytic leukemia (**APL**), all trans retinoid acid (**ATRA**), Beta thalassemia major (**BTM**), beta thalassemia intermedia (**BTI**), classical Hodgkin lymphoma (**cHL**), Chronic myeloid leukemia (**CML**), Diffuse large B cell lymphoma (**DLBCL**), Essential thrombocythemia (**ET**), Hodgkin lymphoma (**HL**), Non-Hodgkin lymphoma (**NHL**), high-performance liquid chromatography (**HPLC**), Multiple myeloma (**MM**), myeloproliferative neoplasm (**MPN**), next generation sequencing (**NGS**), non-transfusion-dependent thalassemia (**NTDT**), transfusion-dependent thalassemia (**TDT**), variant allele frequency (**VAF**), Ventimask (**VM**).
